# A multi-site feasibility study for personalized medicine in canines with Osteosarcoma

**DOI:** 10.1186/1479-5876-11-158

**Published:** 2013-07-01

**Authors:** Noel R Monks, David M Cherba, Steven G Kamerling, Heather Simpson, Anthony W Rusk, Derrick Carter, Emily Eugster, Marie Mooney, Robert Sigler, Matthew Steensma, Tessa Grabinski, Keith R Marotti, Craig P Webb

**Affiliations:** 1 Center for Translational Medicine, Van Andel Research Institute, 333 Bostwick Ave NE, Grand Rapids, MI 49503, USA; 2 Zoetis Inc. (formerly Pfizer Animal Health), Kalamazoo, MI 49007, USA; 3 Animal Clinical Investigation, LLC, Washington, DC 20016, USA; 4 Research Essential Services, LLC, Plymouth, MI 48170, USA; 5 Laboratory of Musculoskeletal Oncology, Van Andel Research Institute, Grand Rapids, MI 49503, USA

**Keywords:** Personalized medicine, Feasibility trial, Canine, Osteosarcoma, Gene expression, Drug prediction

## Abstract

**Background:**

A successful therapeutic strategy, specifically tailored to the molecular constitution of an individual and their disease, is an ambitious objective of modern medicine. In this report, we highlight a feasibility study in canine osteosarcoma focused on refining the infrastructure and processes required for prospective clinical trials using a series of gene expression-based Personalized Medicine (PMed) algorithms to predict suitable therapies within 5 days of sample receipt.

**Methods:**

Tumor tissue samples were collected immediately following limb amputation and shipped overnight from veterinary practices. Upon receipt (day 1), RNA was extracted from snap-frozen tissue, with an adjacent H&E section for pathological diagnosis. Samples passing RNA and pathology QC were shipped to a CLIA-certified laboratory for genomic profiling. After mapping of canine probe sets to human genes and normalization against a (normal) reference set, gene level Z-scores were submitted to the PMed algorithms. The resulting PMed report was immediately forwarded to the veterinarians. Upon receipt and review of the PMed report, feedback from the practicing veterinarians was captured.

**Results:**

20 subjects were enrolled over a 5 month period. Tissue from 13 subjects passed both histological and RNA QC and were submitted for genomic analysis and subsequent PMed analysis and report generation. 11 of the 13 samples for which PMed reports were produced were communicated to the veterinarian within the target 5 business days. Of the 7 samples that failed QC, 4 were due to poor RNA quality, whereas 2 were failed following pathological review. Comments from the practicing veterinarians were generally positive and constructive, highlighting a number of areas for improvement, including enhanced education regarding PMed report interpretation, drug availability, affordable pricing and suitable canine dosing.

**Conclusions:**

This feasibility trial demonstrated that with the appropriate infrastructure and processes it is possible to perform an in-depth molecular analysis of a patient’s tumor in support of real time therapeutic decision making within 5 days of sample receipt. A number of areas for improvement have been identified that should reduce the level of sample attrition and support clinical decision making.

## Background

The treatment of cancer is constantly evolving towards the integration of ever advancing knowledge of disease processes and improvements in molecular and computational technologies. Until recently, approaches towards the treatment of cancer have been disease centric and predominantly determined on the basis of histological classification [1-3]. However, the disparate responses of patients to a given agent with “the same” disease defined through this method of nosology has been attributed to significant molecular heterogeneity within phenotypically defined tumors, and demands the inclusion of molecular biomarkers towards the improved classification of cancers [4,5].

The last 20 years has seen an explosion in both genomic and proteomic technologies, that have assisted in increasing our understanding of disease heterogeneity and have aggressively driven the focus of both drug discovery and development towards the fundamental molecular drivers of disease. Advances in molecular and computational technologies now permit the global analysis of the genome, epigenome, proteome and metabolome at unprecedented granularity, and provide opportunities to study disease heterogeneity within an individual and across populations. This revolution in technologies has made the promise of personalized medicine a reality, through which health care can be customized/tailored for an individual based on information derived from the patient and/or their disease [6]. In the sub-branch of personalized medicine often referred to as pharmacogenomics or precision therapeutics, molecular biomarkers are being used with increased frequency to identify agents with predicted efficacy (and/or reduced toxicity). Oncology is driving the adoption of PMed, where examples include the recommended administration of trastuzumab for tumors exhibiting HER-2 receptor gene amplification or protein over-expression, tamoxifen in breast cancers overexpressing the estrogen receptor, imatinib in the treatment of AML harboring the BCR-ABL translocation [7], and Vemurafinib in the treatment of melanomas carrying the BRAF V600E/K mutation [8]. In addition to these relatively simple drug-single biomarker rules, gene/protein panels are increasingly being use in the diagnostic/prognostic setting to identify patients that would best benefit from neoadjuvant or adjuvant therapy [9-12]. Germline determinants of drug response in key drug metabolism enzymes such as CYP450 have also been identified and are being assessed for their ability to optimize the therapeutic index of agents in the clinic [13]. Such examples are a clear indication that the field of oncology is moving towards rational selection of appropriate therapies for individual patients. However, these tests are limited in that they do not provide global coverage of the genome, and are restricted to a handful of select agents and cancer types. It is clear that a more comprehensive and systematic approach is required to maximize the utility of new genomic and computational technologies and expand drug coverage, and thereby more rapidly and broadly advance the implementation of precision therapy in oncology.

Optimization of PMed through human clinical trials is challenging as refinement of these methods is frequently muddied against a background of standard of care therapy and therapeutic refractoriness. Preclinical mouse models, although offering the advantages of low cost, accelerated endpoints, and ease of genetic manipulation are far from adequate [14]. Human cancers arise spontaneously and are polygenic involving coordinate networks of genes that evolve over time, whilst transgenic mouse models primarily involving the modulation of one or two genes to drive rapid onset malignancies. The classical human cell line-xenograft mouse model used predominantly in drug development typically requires an immune compromised background, thus eliminating the influence of a syngeneic environment in the development of the disease. The emergence of tumorgraft (patient-derived tumor xenografts) models has advanced the field of *in vivo* cancer models due to reduced genetic drift, persistence of human tumor heterogeneity, and maintenance of the tumor microenvironment [15,16]. However, these models also typically require immune compromised mice and are sub-optimal when compared to spontaneously arising cancers in non-laboratory subjects.

To address the void between preclinical models and clinical medicine, many researchers have increasingly turned to comparative oncology as an alternative clinical model of human disease. Comparative oncology describes the study of spontaneous cancers in non-human species, most frequently referring to those animals that are considered pets/companions [17,18]. Canines, in particular, have rapidly risen to become a favored model for the study of human disease with around 400 inherited diseases that have cognate human conditions [19]. Studies have shown that canines are far superior models of human cancers than rodents, being more similar histologically and molecularly at the levels of both DNA and protein sequence [20]. Stark similarities in the molecular drivers of disease, including oncogenes, tumor suppressors and mutations have all been shown to contribute to the development of cancer in both dogs and humans [21]. Additional factors in favor of the selection of canines as a translational model include a shared environment, the contribution of etiological factors including nutrition, age and sex, and analogous diagnostic and interventional procedures used in veterinary and human healthcare (reviewed in [17,18,22]). Genetically, canines are ideal candidates to study the fundamental genetic drivers of human disease, owing to the breed specific proclivity of particular cancer types. This phenomenon has arisen following approximately 200 years of inbreeding, restricting the genetic flow between breeds, consequently selecting for founder mutations that are associated with breed specific traits and disease [21,23]. Canines age 5-8-times more rapidly than humans, which provides an opportunity to study diseases that are age related [18]. Similarly, and in part due to less aggressive disease management, cancer progression is quicker in dogs, with the average disease-free interval being 18 months compared to 7 years in humans [17]. This has significant benefits as it enables shorter clinical trials, which, alongside similar response to conventional (human) therapeutic regimes, support the use of canine subjects in early clinical trials. The lack of established standard of care treatments for canines also provides an opportunity to evaluate novel therapies and protocols in subjects with less advanced, non-refractory (even naïve) disease, prospects that are difficult to impossible in human patients [17].

Osteosarcoma (OSA) is an ideal disease candidate for inter-species investigation of personalized medicine approaches. It has been shown that canine and human OSA are analogous at a number of levels, histologically, behaviorally, genetically and with regards to response to therapy (reviewed by [18,22,24]). The incidence of OSA in dogs is 20-fold greater than in humans [25], with around 10,000 canines diagnosed per year compared to approximately 2,650 primary bone tumors in humans (a statistic which includes OSA, chondrosarcoma, Ewings sarcoma and malignant fibrous histiocytoma) [18], therefore increasing the number of subjects that are available for recruitment into clinical trials. OSA occurs primarily at around 7–9 years of age [26], with large and giant breeds (e.g. Saint Bernards Greyhounds, Great Danes, German Shepherds, Golden Retrievers) having a 60-fold greater risk of developing OSA [27,28]. Following amputation alone, >90% of dogs die within a year, with cause of death being related to the development of metastasis, typically to the lung [26]. Adjuvant chemotherapy can further improve survival from 103–175 days following surgery alone, to 262–450 days (review – [24]). Even considering these dramatic changes in survival time, the long-term prognosis for OSA is morose and 2 year survival has been measured at between 10-26% [24]. It is the poor long term survival of canines with OSA, along with the translational value for the corresponding human disease, which makes this tumor an ideal candidate for the identification of novel therapeutic agents using PMed approaches.

In this report we outline the results of a 20 subject feasibility study in canine osteosarcoma, with the key goal of establishing the infrastructure and logistics for a subsequent prospective large scale PMed trial. The design of this study was not intended to validate the clinical utility of a PMed report in dogs with cancer. We describe the utility of global gene expression profiling of osteosarcomas from canine patients, which in parallel with advances in laboratory procedures, bioinformatics tools and a physician reporting interface permits the application of real-time genomic medicine in the context of veterinary medicine. Gene expression profiling is a tool that has been used by other groups to examine canine osteosarcoma to identify differentially expressed genes that can stratify patients as short or long-term survival [29] and identify biomarkers and pathways associated with patient prognosis [30].

The PMed system utilized here is an assembly of 5 predictive methodologies that rank the overall drugs predictions weighted by the number of methods which predict the drug, frequency of inclusion (multiple targets for a drug) and strength of prediction (high differential expression above the normal reference). The PMed system is drug centric and focused around 183 FDA approved medications (see Additional file 1: Table S1). Drug target expression [31] and drug sensitive/resistant Biomarker rules [32] are both linked directly to the expression levels of individual genes. Two of the methods namely Drug sensitivity signatures – PGSEA [33] and Drug Response Signatures-CMAP [34], use global gene expression patterns which have been associated with drug effectiveness. The fifth method uses a global gene-gene interaction database, to identify putative drug targets based on topological analysis of differentially expressed genes that are up or downstream of transcriptional events (Network Target activity [35]). Global gene expression analysis (microarray) has been used in both a clinical trial where it was shown to benefit progression free survival [32] and *in vitro* to better predict pharmacological response [36].

Within this investigational trial, we established a number of objectives including; a) to establish a timely process for the collection, shipping, processing and diagnosis of tumor samples from canine osteosarcoma patients, and; b) to determine the feasibility of generating a PMed report from predictive modeling of canine tumor-derived gene expression data within 5 business days of sample receipt. Although this study was not designed to include a treatment arm, we collected the opinions of the practicing veterinarians regarding the potential clinical utility of the PMed report. We identified that, while the presentation of the PMed report to the veterinarian in a timely fashion is critical to support the clinical management of the disease, the interpretation and implementation of the report by the clinician is essential for the success of PMed Trials and clinical adoption in the future.

## Methods

### Study overview

**Figure 1:**
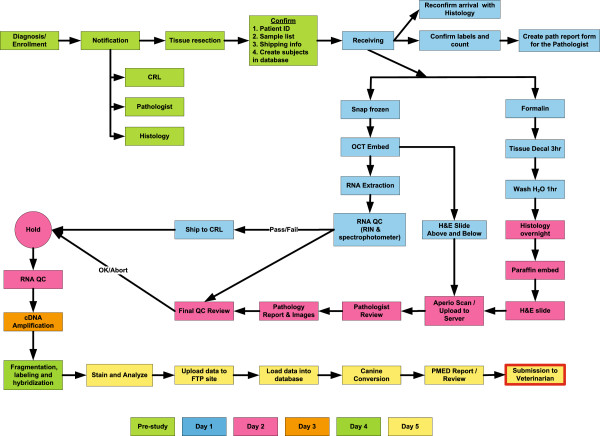
**Process diagram defining the steps and timing of the osteosarcoma PMed feasibility study.** The figure highlights the numerous steps, which are grouped by day (color), that were followed in order to produce a PMed report in 5 business days from the receipt of the sample.

The study design and overall processes are summarized in Figure 1. In brief, the study involved the identification and recruitment of 20 mixed breed or purebred dogs with suspected appendicular OSA. The MiniMax approach was used to identify 20 patients as a suitable sample size for this study. Based on previous work the expected censor rate due to sample quality and diagnosis was conservatively estimated at 25% (5 patients). In this regard an initial patient population of 20 patients provided sufficient power for statistical significance if 70% of the samples (11 of 15) were completed in the 5-day targeted time limit. The presumptive diagnosis of OSA was based on review of limb radiographs or from histology performed pre-study. Study dogs were screened and samples collected at Animal Clinical Investigation (ACI) network clinics. Ethical approval for this study was granted by the respective Institutional Animal Care and Use Committees (IACUC) at the Van Andel Research Institute (VARI), Zoetis and Animal Clinical Investigations (ACI). All procedures and veterinary care provided to each patient was performed by trained veterinary specialists who followed standard clinical or protocol driven procedures as documented in the study documentation training files. For those patients that met the inclusion criteria, the amputation surgery was scheduled at which time the tumor collection was performed. The appropriate samples were sent to VARI, processed and submitted to Clinical Reference Laboratory (CRL) for genomic profiling. Following receipt of the Affymetrix gene expression data from CRL, it was imported into VARI’s database and input into the PMed analysis/reporting system. The PMed report was then distributed to ACI, who further distributed to the collection sites and obtained responses to a questionnaire regarding the clinical utility of the provided PMed report.

### Study eligibility

Once written owner consent had been obtained by the clinical site, inclusion/exclusion criteria, patient demography and physical exam data were evaluated by the investigator. If not previously performed, diseased limb radiographs and interpretation (required) and abdominal/thoracic radiographs (if determined necessary by the Investigator) were performed, evaluated and results recorded. Patients that met the study inclusion criteria (Table 1) were enrolled in the study and amputation scheduled.

**Table 1:** Study inclusion and exclusion criteria

**Inclusion criteria**	**Exclusion criteria**
Client-owned pet dogs ≥ 1 year of age, any gender and weight.	Anticipated poor owner compliance.
Informed owner consent prior to enrollment screening.	Pregnant or likely to become pregnant.
Suspected diagnosis of primary appendicular OSA (+/− metastasis) via radiographs and physical exam or confirmed diagnosis based cytology or histopathology of the affected limb.	Receiving/have received treatment for cancer including chemotherapy, bisphosphonate therapy, prednisone therapy, radiation therapy or immunotherapy, other than Non-steroidal Anti-inflammatory Drugs (NSAIDS).
Any disease stage.	Previously amputated limb as a result of OSA.
Naïve (i.e. *de novo*) cases.	Concurrent malignancy that is non-appendicular OSA.
Planned amputation.	Primary cancer originated at an anatomical sites other than appendicular OSA.
	Other serious systemic disorder incompatible with this study.
	Acupuncture treatment less than 2 weeks prior to sample collection day (dogs that have commenced acupuncture > 2 weeks prior to Day 0 can be included but they must stay on their acupuncture treatment during the study period).
	Dogs that are participating in another clinical trial, (subjects are allowed to enroll on a trial after the samples have been collected).
	Quality Control (QC) failure (histopathological or genomic).

### Site training

Prior to the start of the study, training was provided to each of the participating veterinary practices with regards to the sampling of the tumor from the amputated limbs. Specific instructions were provided as to where best to harvest tumor tissue to minimize normal tissue contamination and bone involvement which could complicate downstream procedures, e.g. cryo-sectioning of snap frozen tissue for RNA extraction and H&E sectioning. The primary site of harvest was designated as grossly viable, unmixed tissue present at the advancing front of the tumor. This site was commonly identified outside of the marrow space, and was evident in the majority of cases. The medullary cavity was identified as the secondary site of harvest.

### Normal tissue reference

The purpose of the reference set is to facilitate comparative analysis of the fundamental differences of the tumor versus a biologically appropriate denominator, i.e. normal tissue capturing the cell(s) of origin of the tumor. Due to the practical challenges in obtaining breed, age and sex matched disease-free normal bone in a study that was open to all breeds, combined with the need to have the reference set available prior to the start of enrollment, five total bone samples (cortical bone and marrow) were collected from 5 disease free Beagles. These five normal samples were processed identically to the tumor samples in order to provide a tissue specific reference for the tumor samples. Standard statistics consisting of mean and standard deviation were compiled for each probe set across the reference samples. The tumor sample probe sets intensities were transformed to Z-Score by utilizing the reference sample probe set mean and standard deviation. This score was subsequently utilized by the PMed methods to suggest possible therapies.

### Tumor harvest

Following determination of eligibility and obtaining owner informed consent, amputation surgery was scheduled. Radiographs of the diseased limb were collected prior to amputation to guide the best site for tumor harvest. At the time of the surgery, immediately post amputation of the limb, up to 5 tumor specimens (measuring approximately 4 mm^3^ each) were obtained per patient. Sample requirements and prioritizations are described in Table 2. Samples numbered 1–3 were mandatory collections, whereas samples numbered 4–5 were optional and only collected if excess representative tumor tissue was available. Sample 1 (formalin fixed) was sent to the collection sites’ pathologist as per normal practice for diagnostic evaluation. Samples 2–5 were shipped immediately to VARI using priority overnight delivery; snap frozen samples were shipped on dry ice, whereas the formalin fixed tissue was shipped on −20°C ice packs.

**Table 2:** Sample prioritization and procurement

**Sample #**	**Preparation**	**Purpose**	**Required**	**Destination and shipping conditions**
**1**	Formalin	Confirmatory histological diagnosis	Yes	Site selected diagnostic pathology
**2**	Snap Frozen	Genomic profiling (PMed) and Histological diagnosis^**1**^	Yes	VARI **(dry ice)**
**3**	Formalin	Histological diagnosis^**2**^	Yes	VARI **(wet ice)**
**4**	Snap Frozen	Back up/tissue banking	If tissue is available	VARI **(dry ice)**
**5**	Formalin	Backup/tissue banking	If tissue is available	VARI **(wet ice)**

^**1**^ Assessment of tumor content (% tumor by nuclei, % normal tissue by nuclei, % necrosis).

^**2**^ Back-up source for assessment of tumor content if no sections available from Snap frozen tissue.

### Tissue processing

Upon receipt at VARI, the samples were logged and processed immediately. For the purpose of this study, Day 1 was considered to be the time at which tissue processing (RNA isolation and pathology) commenced. In the case where samples were received on a Friday (or on the weekend), day 1 automatically defaulted to the next business day. When sample processing was delayed, formalin fixed samples were transferred to 70% ethanol (to avoid over fixation) and stored at room temperature (RT), while snap frozen samples were stored at −80°C.

### Snap frozen

The snap frozen tissue was maintained on dry ice and immediately embedded in Optimal Cutting Temperature (Tissue-Tek® O.C.T.) media, which was used to hold the tissue in position during cryosectioning. When possible, two 5 μM sections above and below preparative RNA cuts were taken (mounted together on a single slide) and stained with hematoxylin and eosin (H&E). The H&E slides were scanned using an Aperio ScanScope XT (Aperio, Vista, CA.), and uploaded to a centralized location for evaluation by an off-site veterinary pathologist. In between the H&E sections, 8-10x 50 μM slices were collected for RNA extraction.

### Formalin fixed

Due to the production of mineralized osteoid by osteosacrcomas which can hinder the sectioning of the tissue, the formalin fixed sample was decalcified for 3 hours in Formic-Decal solution (Rowley Biochemical, MA), followed by a 1 hr. wash in running tap water. The tissue was subsequently processed overnight into paraffin, embedded, sectioned and stained with H&E. These slides were also scanned using the Aperio ScanScope XT and the image uploaded to a centralized location for review by the veterinary pathologist.

### RNA extraction

Total RNA was isolated directly from the OCT sections using Trizol, with an initial homogenization for 2 minutes at 40 oscillations/second using the TissueLyser LT (Qiagen), followed by a DNase digestion and RNA clean-up using Qiagen RNeasy Mini Kit. Following elution in H_2_O, the RNA was analyzed spectrophotometrically (Nanodrop) to determine RNA yield and purity (A260/280). RNA integrity was subsequently determined using the Agilent RNA 6000 Nano Kit on the Agilent Bioanalyzer 2100 (Agilent Technologies, Inc. CA.). In order for samples to proceed to Affymetrix GeneChip profiling, three RNA QC parameters had to be met; RNA yield >20 ng, A260/280 ≥1.8, and an RNA Integrity Number (RIN) ≥ 6.0. The RNA integrity number is generated by an algorithm which uses the entire electrophoretic trace of the RNA sample, rather than just the ribosomal bands, to assess the presence or absence of degradation products. A RIN is calculated by the software that interprets a sample’s RNA electropherogram, independent of concentration, and assigns a number between 1 (highly degraded) and 10 (intact) [37]. Samples that passed these criteria were immediately shipped overnight on dry ice to a CLIA (Clinical Laboratory Improvement Amendments) certified external contract laboratory (CRL). Samples failing any of these QC parameters were not sent for pathological review and were censored from the study and classified as a fail.

### Pathological assessment and diagnosis

The primary pathological assessment was made using the H&E sections from the OCT embedded snap frozen tissue, taken immediately adjacent (above and below) to the 50 μM sections used for the RNA extraction. In the event that the OCT H&E section were unavailable (e.g. the tissue couldn’t be cut at 5 μM sections) the H&E section from the FFPE tissue (derived from the same tumor mass) was used. In either case, the pathologist was provided with H&E images from both sample types, in the event that the snap frozen H&E section was not of sufficient quality to make a clear diagnosis and determination of tissue composition. The tissue sections were assessed for % viable tumor (by Nuclei), % viable normal tissue (by nuclei) and % Necrosis; to pass QC these values needed to be ≥50%, <50% and ≤20%, respectively. Failure of any of these QC parameters resulted in censoring from the study and classification as a fail.

### Gene expression analysis

Upon receipt of the sample at the CLIA Certified Laboratory the following day (Day 2), the RNA samples were held and processing delayed until the results of the pathological assessment were available. Samples, that passed pathological QC, were then subject to a second RNA QC (as described above) as required by the CLIA laboratory Standard Operating Procedure (SOP) to ensure no loss in RNA integrity during shipment and thaw. Fifty nanograms of total RNA was used for cDNA synthesis and amplification using the NuGEN Ovation Pico WTA System (NuGEN Technologies, Inc. CA), following which additional cDNA QC checks were performed (total cDNA yield ≥5 μg, A260/280 ≥1.8). Following fragmentation and labeling (NuGEN Encore Biotin Module) the cDNA was hybridized overnight to the Affymetrix Canine 2.0 array. The arrays were then washed, labeled (GeneChip Hybridization, wash and stain kit, Affymetrix, Inc. CA.) and scanned using the Affymetrix gene chip scanner 3000 7G. The image file produced by the scanner was subsequently analyzed using the Affymetrix Expression Console producing .CEL and .DAT files, from which additional post-analysis QC pass/fail criteria were recorded, including, background (<100), percent present (>30%), scale factor (<100), spiked controls: 3’ signal (bioB < bioC < bioD < cre) and a visual inspection of the image file for surface anomalies. Post-analysis QC failures would result in the data not being submitted for PMed report generation. Upon passing all criteria, a MAS5.0 normalization process was performed producing a tab delimited pivot table with probe identifiers, quality scores, present calls, and intensities. All quality information and data files along with the original image files were uploaded to a secure FTP site hosted at VARI.

### Bioinformatics and PMed report generation

The overall PMed system developed at VARI has been described in detail elsewhere [38,39]. The iteration of the system used for this study leverages several published methodologies (see below) that attempt to identify biopharmaceutical agents/natural products with predicted efficacy on the basis of differentially expressed genes (DEGs) in the sample(s) of interest. Each individual method uses a series of assumptions, and each has the capacity to predict the efficacy of a defined number of agents (with some overlap between methods). For this study, only agents approved by the FDA for human use (in any disease indication) were included. Additional file 1: Table S1 lists the 183 agents that could have been predicted by at least one method in this study, along with information on canine dosing if known at the duration of the study. The input to all methods is the normalized Z-score for a given Affymetrix probe set which, as described above, represents the expression of a gene in the OSA sample in terms of the number of standard deviations from the mean in the reference sample set (normal bone).

The initial step for processing each canine array is to convert the probe set intensities for each tumor sample to Z-scores using the reference set statistics (see above). A Z-score (or standard score) is a numerical value that indicates how many standard deviations a data point is above or below the mean of the whole data set. Since the PMed system was built on the basis of the human Affymetrix GeneChip, a key step in the process was the conversion of canine Affymetrix Z-score data to the human counterpart. This was achieved by initial mapping the Affymetrix GeneChip data to canine Entrez Gene version 21 annotation. In the cases where multiple probe sets mapped to the same gene they were aggregated using the arithmetic mean to a single value for the corresponding canine Entrez Gene identifier. The canine Entrez gene identifiers were then converted to human Entrez Gene homolog using the National Cancer Institute’s Homologene database (dated 11/15/2010). Any canine Entrez Genes that could not be concisely mapped to their human homolog were removed. Finally, the human Entrez Gene identifiers (preserving the canine Z-score data) were mapped to the appropriate Affymetrix U133 2.0 plus probe set ID using the Affymetrix U133 2.0 plus annotation version 31 data file.

#### Biomarker rules

This method uses simple binary logic biomarker rules to indicate or contraindicate specific agents [32]. The biomarker rules are established on the basis of vetted literature and compiled in a database in the simple form: IF biomarker expressed > or < predefined Z-score value THEN DO or DO NOT recommend drug. While each biomarker-drug rule can be weighted on the basis of the disease context of published findings, the iteration of the system used in this study assumed equal weighting for all biomarker rules irrespective on disease context (e.g. a biomarker rule established in the context of lung cancer (e.g. ERCC1 as a marker of cisplatin resistance) would be utilized in this feasibility study).

#### Drug target expression

This is analogous to the biomarker rules approach described above except that it relies exclusively on the known mechanism of action of each agent, and does not require well vetted literature to demonstrate an association between the expression of the drug target and the drug’s efficacy. This method utilizes a human drug-target (mechanism of action) knowledge base developed from various sources including DrugBank [31], MetaCore (Thomson Reuters / GeneGo), MedTrack, PharmGKB, UpToDate and DrugDex (Thomson Reuters). In this study, drug targets found to be over-expressed (based upon a Z-score threshold of ≥ +3) in a patient’s tumor relative to the reference set were identified along with the agent that inhibits the targets activity.

#### Drug response signatures

The Connectivity Map concept was initially developed by the Broad Institute in an attempt to connect molecular signatures of disease with drug-induced changes in gene expression [34]; drugs that are shown to induce changes in gene expression in a set of cancer cell lines which reverse the disease-associated DEG’s towards normal levels are identified as therapeutic candidates. In our study, the maximum number of DEG’s submitted to this algorithm were capped at 500 (the Z-score threshold for this method was set to ≥ + 2.0 or ≤ −2.0) and the method used rank-based statistics to identify candidate drugs as described previously [34].

#### Drug sensitivity signatures

This method adopts Parametric Gene Set Enrichment Analysis (PGSEA) using the NCI-60 cell line drug sensitivity signatures [33]. Gene expression signatures associated with differential response to specific drugs on the basis of the NCI 60 cell line *in vitro* drug screen are compared to the tumor-derived gene expression signature. This approach is consistent with well-published methods for inferring drug sensitivity utilizing the NCI-60 cell line dataset and baseline gene expression signatures [40-42].

#### Network target activity

This method predicts the activity (vs. expression) level of drug targets on the basis of a specific type of molecular network analysis referred to as topological analysis which has been described previously [35]. It utilizes the DEG list and pre-requisite knowledge of protein-protein interactions within the knowledge-base of MetaCore (Thomson Reuters/GeneGo) to build complex networks and predict upstream target activity on the basis of observed downstream transcriptional events.

#### PMed report generation

Each of the methods summarized above produces a p-value which is used to score and rank the predicted efficacy of identified agents within each methodology. In addition, a summated drug score (sum of – log (*p*)) was provided as a means to further rank potential agents, along with additional evidence supporting the potential use of the agent in the context of the patient’s disease. For example, current clinical trials and literature evidence identified through an automated search of the disease context (“osteosarcoma”) and the identified drugs were compiled within the PMed report and provided as a further means to select viable agents. The compiled interactive PMed report was then distributed via PDF format to ACI and the enrolling veterinarian. An example of a PMed report provided during the course of this study (for Subject TL-141) is provided in Additional file 2 (PMed Report TL-141).

## Results

The study accrual time for the enrollment of the 20 subjects was 5 months (first biopsy AH-301 - 6/10/2011 to final biopsy RB-187 - 11/10/2011). Table 3 highlights the patient demographics and the dates of enrollment (date of biopsy) for all 20 subjects. The main objective of the study was to assess feasibility in the distribution of a subject-tumor specific PMed report in 5 business days from receipt of the sample. As highlighted in Figure 1, the logistics of this study involved multi-site participation and close monitoring of all aspects of the process including, sample shipping, tissue processing, pathological assessment, gene expression profiling, data management and bioinformatics. Numerous QC criteria were included throughout the study, to monitor the quality of both the samples and the data generated with the goal of providing the highest quality data as input into the PMed system. The VARI generated RNA and pathology QC for all subjects is shown in Table 4. The site specific pathology is also presented in Table 4, although these diagnoses were not part of the pathological QC as the turnaround time for routine clinical samples was frequently greater than 7 days, and thus insufficient within the time restraints of the study goal.

**Table 3:** Patient demographics

**Enrollment order**	**Patient ID**	**DOB**	**Sex**^**1**^	**Breed**	**Date of biopsy**	**Disease location**	**Medullary or Extramedullary**
**1**	AH-301	9-Jun-06	MC	Belgian Malinois	10-Jun-11	Right Distal radius	Extramedullary
**2**	FS-201	19-Aug-02	MC	German Short-Hair Pointer	20-Jun-11	Right Proximal Femur	Medullary
**3**	RV-281	1-May-03	M	Coonhound	21-Jul-11	Left Proximal Humerus	Medullary
**4**	MH-101	27-Jul-04	FS	German Shepherd	2-Aug-11	Right Distal Tibia	Extramedullary
**5**	TL-141	1-Aug-05	MC	Standard Schnauzer	17-Aug-11	Right Distal Femur	Extramedullary
**6**	RB-181	3-Aug-00	FS	Labrador Retriever	19-Aug-11	Right Proximal Tibia	Medullary
**7**	RB-182	1-Jan-03	MC	German Shepherd	01-Sep-11	Right Distal tibia	Not determined
**8**	RB-183	1-Apr-99	FS	Golden Retriever Mix	3-Sep-11	Right Distal Femur	Medullary
**9**	VS-121	13-Feb-09	FS	Golden Retriever	6-Sep-11	Right Proximal Tibia	Extramedullary
**10**	AZ-221	20-Aug-06	FS	Greyhound	7-Sep-11	Right Proximal Humerus	Not determined
**11**	RV-282	2-Jan-04	MC	Labrador Retriever	8-Sep-11	Right Proximal Humerus	Medullary
**12**	NC-161	13-Sep-01	FS	Golden Retriever	13-Sep-11	Left Distal Radius	Extramedullary
**13**	FS-202	16-Sep-99	FS	Greyhound	24-Sep-11	Right Proximal Humerus	Medullary
**14**	FS-203	3-Apr-02	FS	Labrador Retriever	29-Sep-11	Right Proximal Humerus	Extramedullary
**15**	RB-184	23-May-04	FS	Great Dane	1-Oct-11	Left Proximal Tibia	Medullary
**16**	RB-185	20-Aug-00	FS	Greyhound	5-Oct-11	Left Proximal Humerus	Medullary
**17**	RB-186	4-Mar-04	MC	Lab Mix	10-Oct-11	Left Distal Ulna	Both*
**18**	RV-283	22-Apr-04	FS	Great Dane	20-Oct-11	Right Distal Femur	Extramedullary
**19**	NC-162	24-May-04	M	German Shepherd	31-Oct-11	Right proximal Femur	Medullary
**20**	RB-187	3-Nov-04	M	Rottweiler	10-Nov-11	Right Distal Ulna	Both*

^1^ Sex – M – Male; MC - Male castrated; FS – Female Spayed.

* Both indicates cases in which medullary and extramedullary locations were sampled.

**Table 4:** VARI RNA and pathological QC

**Patient ID**	**RNA QC**		**Pathology QC**				
	**A260/280**	**RIN**	**% Viable tumor**^**1**^	**% Viable non-Neoplastic tissue**^**1**^	**% Necrotic tissue**	**VARI diagnosis**	**Clinical site diagnosis**
			**(1st Cut / 2nd Cut)**				
**AH-301**	2.27	8	75/75	5/10	20/15	Chondrosarcoma	Chondrosarcoma
**FS 201**	2.08	7.8	80/80	20/20	15/25	Poorly differentiated tumor. Atypical Osteosarcoma	OSA
**RV 281**	2.11	9.3	75/75	20/20	10/10	Osteosarcoma	Chronic suppurative inflammation
**MH 101**	1.84	5.5^**2**^				No Pathology due to RNA QC failure	OSA
**TL-141**	2.11	6.8	60/50	30/50	10/0	Osteosarcoma.	OSA
**RB-181**	2.11	7.5	80/85	10/5	10/10	Osteosarcoma	OSA
**RB-182**	2.07	8.5	60/60	20/30	20/10	Osteosarcoma	OSA
**RB-183**	2.09	6.8	90/80	10/10	0/10	Osteosarcoma (FFPE)	OSA
**VS 121**	2.06	6.4	90/95	5/0	5/5	Osteosarcoma with a differential diagnosis of undifferentiated sarcoma.	OSA
**AZ-221**	2.09	8.1	50/50	50/40	0/10	Osteosarcoma (FFPE)	OSA
**RV-282**						Sample lost due to shipping delay.	OSA
**NC-01**	2.1	8	80/80	15/10	5/10	Osteosarcoma.	OSA
**FS 202**	2.09	8.5^**3**^	60/65	30/25	10/10	Osteosarcoma. (Frozen and FFPE)	OSA
**FS 203**	2.03	7.2	No evidence of tumor, values not recorded	No evidence of tumor (Frozen and FFPE)	Chronic inflammation		
**RB 184**	2.15	6.8^**4**^	65/70	30/30	5/0	Osteosarcoma.	OSA
**RB 185**	1.8	6.9	90/75	5/20	5/15	Osteosarcoma. (FFPE)	OSA
**RB 186**	2.11	5.4^**2**^				No Pathology due to RNA QC failure	OSA
**RV 283**	2.09	8.4	60/60	40/40	0/0	Undifferentiated sarcoma (FFPE).	OSA
**NC 162**	2.08	7.4	0/0	70/70	30/30	Normal tissue.	Not available
**RB 187**	2.09	8.6^**3**^	65/65	25/25	10/10	Osteosarcoma	Suspected Synovial carcinoma

^**1**^ - Scores based on % nuclei.

^**2**^ - Failed RIN.

^**3**^ No RIN generated by Agilent software, even though sample visually looks good. Altered Fast lane threshold (to 1) to obtain RIN.

^**4**^ Sample shipped without VARI RIN analysis, value given determined at CRL.

**Figure 2:**
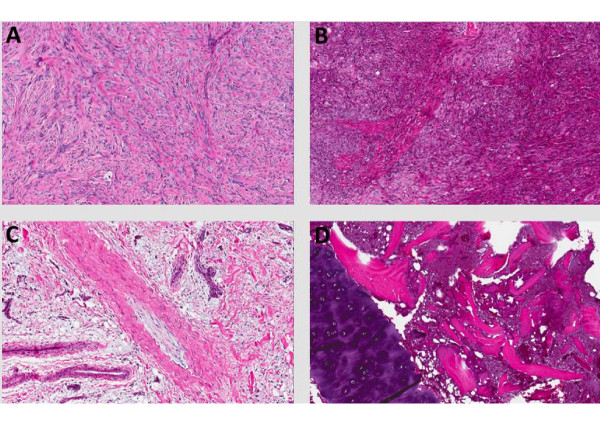
**Representative images taken from FFPE sections of subjects enrolled in the study. A** (RB-181) and **B** (RB-183) highlight classical osteosarcoma displaying multinucleated tumor osteoblast and osteoid synthesis. The histologies shown in Figures **C** and **D** (FS-203 and NC-162, respectively) correspond to samples that failed VARI pathology QC as they failed to show evidence of a suitable proportion of neoplastic disease.

Of the 20 subjects recruited onto this study, 7 failed QC and were not profiled. None of the samples that were submitted for expression profiling failed post-array QC assessment. Table 5 lists the subjects that failed QC and provides the details for their exclusion. RIN failure at both VARI and/or CRL accounted for attrition of 4/7 samples. In addition, 2/7 samples failed pathological QC, whereas 1/7 samples was lost due to a shipping error from the clinical site. In the cases of samples that passed RIN QC but failed VARI Pathology QC (e.g. FS-203 and NC-162 – see Figure 2), the external contract laboratory was immediately notified and Affymetrix profiling aborted. VARI was also notified by the external contract laboratory if samples failed their RNA QC. If the external contract laboratory RIN QC was below 6.0 then upon consultation with VARI, Affymetrix processing was aborted. In the two cases where no RIN could be generated (i.e. an error was flagged by the Agilent software (see note ^**3**^ in Table 4)) but the electropherogram passed visual inspection (e.g. FS-202 and RB-187), VARI provided the go ahead to proceed with Affymetrix gene expression profiling. In each of these two cases the error was due to an unexpected peak in the fast lane which, upon changing this threshold to 1 (this was only performed at VARI, since due to the CLIA SOP, CRL was not able to change the analysis settings of the Agilent Bioanalyzer), resulted in a calculated RIN that was in excess of 8.

**Table 5:** Samples that failed QC and reason for exclusion from the PMed analysis

**Patient ID**	**Reason for exclusion from study**
**MH-101**	Failed RIN at VARI (5.5) ^**1**^
**RB-183**	Failed RIN at CRL (no score generated)
**RV-282**	Samples shipped 9/8/2001 overnight, scheduled to arrive on Friday 9/9/2011. Actual arrival 9/12/2011. Dry ice evaporated and sample was at RT.
**FS-203**	Failed Pathology QC (No neoplastic tissue observed).
**RB-185**	Failed RIN at CRL (5.8) ^**1**^
**RB-186**	Failed RIN at VARI (5.4) ^**1**^
**NC-162**	Failed Pathology (No neoplastic tissue observed)

^**1**^ All samples submitted for GeneChip analysis passed RIN at both VARI and CRL.

**Figure 3:**
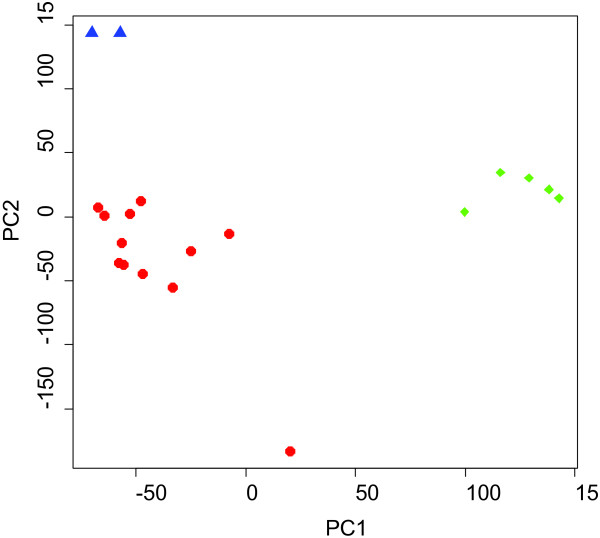
**Principal component analysis (PCA).** 43,035 probe sets clearly distinguishing normal bone (green) from OSA samples (red). The biological replicates (VS-121 and VS-01) highlighted in blue and show minimal variance.

Robust and reproducible data is important to studies of this type where samples are handled and analyzed individually. To address the overall data quality, Principal Component Analysis (PCA) was performed on both the 5 normal bone samples and 14 qualified osteosarcoma tumors (Figure 3). A secondary measure to address assay precision included the addition of a biological replicate, identified as VS-01, which was isolated from a second piece of tumor from subject VS-121. PCA analysis clearly reveals a difference between normal and tumor tissue at the level of the 1st and 2nd principle components (the components contributing to the most variance), the 5x total bone harvests from disease-free subjects (green data points) clearly separate from the osteosarcoma tumor tissue (red data points). Additionally, within the same tumor sample, biological replicates (VS-121 and VS-01, isolated from 2 separate pieces of the same tumor) co-cluster tightly (blue data points), demonstrating a high level of reproducibility between the replicates and thus providing confidence that comparative analyses can be made between all data generated in the study. Furthermore, the PCA also reveals the degree of genomic heterogeneity between the different OSA tumor samples, supporting the use of a PMed approach to the selection of suitable therapies on the basis of molecular profiling.

The primary objective for the study was to determine the feasibility of processing a tumor biopsy through to PMed report generation and distribution to the veterinarians in 5 working days. Table 6 displays the data pertaining to the PMed report turnaround time, highlighting the dates of surgery, sample receipt at VARI, shipment of pre-qualified RNA to CRL, data receipt back from CRL, and the date on which the PMed report was distributed (via email) to ACI.

**Table 6:** Details of the study timing and turnaround

**Patient ID**	**Date**					**Turnaround (Days)**		
	**Surgery date**	**Arrival at VARI**	**RNA Shipped to CRL**	**Date data uploaded from CRL**	**PMed Report sent**	**Total days from sample receipt to PMed Report**	**Adjustment Weekends/ Holidays**	**Final (adjusted)**
**AH-301**	6/10/2011 Fri	6/13/2011 Mon	6/13/2011 Mon	6/16/2011 Thu	6/17/2011 Fri	5	0	5
**FS-201**	6/20/2011 Mon	6/21/2011 Tue	6/21/2011 Tue	6/27/2011 Mon	6/27/2011 Mon ^**2**^	7	−2	5
**RV-281**	7/21/2011 Thu	7/22/2011 Fri ^**1**^	7/25/2011 Mon	7/28/2011 Thu	7/29/2011 Fri	7	−2	5
**MH-101**	8/2/2011 Tue	8/3/2011 Wed	**Failed RNA QC (RIN) at VARI**					
**TL-141**	8/18/2011 Thu	8/19/2011 Fri ^**1**^	8/22/2011 Mon	8/25/2011 Thu	8/26/2011 Fri	7	−2	5
**RB-181**	8/19/2011 Fri	8/25/2011 Thu	8/25/2011 Thu	8/31/2011 Wed	8/31/2011 Wed	7	−2	5
**RB-182**	9/1/2011 Thu	9/2/2011 Fri ^**1**^	9/7/2011 Wed	9/13/2011 Tue	9/13/2011 Tue	11	-(4 ^**3**^ + 1 ^**4**^)	6 ^**5**^
**RB-183**	9/3/2011 Sat	9/8/2011 Thu	9/8/2011 Thu	**Failed RNA QC (RIN) at CRL**				
**VS-121**	9/6/2011 Tue	9/7/2011 Wed	9/7/2011 Wed	9/13/2011 Tue	9/13/2011 Tue	7	−2	5
**AZ-221**	9/7/2011 Wed	9/8/2011 Thu	9/8/2011 Thu	9/13/2011 Tue	9/13/2011 Tue	6	−2	4
**RV-282**	9/8/2011 Thu	9/12/2011 Mon ^**6**^	**Sample lost due to shipping error.**					
**NC-161**	9/13/2011 Tue	9/15/2011 Thu	9/15/2011 Thu	9/20/2011 Tue	9/20/2011 Tue	6	−2	4
**FS-202**	9/24/2011 Sat	9/30/2011 Fri ^**1**^	10/3/2011 Mon	10/7/2011 Fri	10/9/2011 Sun	9	−2	7 ^**7**^
**FS-203**	9/29/2011 Thu	9/30/2011 Fri ^**1**^	10/3/2011 Mon	**Failed pathology QC.**				
**RB-184**	10/1/2011 Sat	10/5/2011 Wed	10/5/2011 Wed	10/11/2011 Tue	10/11/2011 Tue	7	−2	5
**RB-185**	10/5/2011 Wed	10/7/2011 Fri ^**1**^	10/10/2011 Mon	**Failed RNA QC (RIN) at CRL**				
**RB-186**	10/10/2011 Mon	10/14/2011 Fri ^**1**^	**Failed RNA QC (RIN) at VARI**					
**RV-283**	10/20/2011 Thu	10/21/2011 Fri ^**1**^	10/24/2011 Mon	10/27/2011 Thu	10/28/2011 Fri	7	−2	5
**NC-162**	10/31/2011 Mon	11/2/2011 Wed	11/2/2011 Wed	**Failed pathology QC.**				
**RB-187**	11/10/2011 Thu	11/16/2011 Wed	11/16/2011 Wed	11/22/2011 Tue	11/22/2011 Tue	7	−2	5

^**1**^ Samples received on Fridays were automatically defaulted to start on Mondays (Day 1).

^**2**^ Report generated manually on Monday June 27th 2011 due to PDF conversion problems within the software. Corrected report sent out the following day (Tuesday June 28th 2011).

^**3**^ Two weekends (4 Days).

^**4**^ Sample processing delayed 1 day (9/5/2011) due to Labor Day holiday.

^**5**^ One day lost due to absence of critical Histopathology staff.

^**6**^ Sample did not arrive on time (due Friday September 9th 2011) arrived thawed on the following Monday (September 12th 2011).

^**7**^ Report not delivered on the Friday (Day 5) due to issues with a link out to an external database. Report successfully completed on the Sunday and submitted.

Day 1 was considered to be the day on which the samples arrived at VARI. There were however two exceptions; those samples received on a Friday, Day 1 was automatically defaulted to Monday (or the next business day). This process was instigated to avoid the shipment of samples to the external contract laboratory into the weekend. 8 of the 20 samples were handled in this manner (see footnote ^1^ in Table 6).

The total turnaround time was calculated as the number of days from the receipt of the sample at VARI to the date on which the PMed report was sent (Table 6). The final turnaround time was adjusted to account for weekends and public holidays. In the majority of cases the PMed reports were released within 5 business days. Two subjects, RB-182 and FS-202, failed this objective. RB-182 was received on a Friday and thus processing was delayed to the next business day, as the following Monday was a public holiday, processing should have started on the Tuesday. However, due to the absence of critical histopathology staff, the start of processing was delayed until the Wednesday. FS-202 was processed and data received on schedule for a 5 day turnaround. However, due to the loss of connectivity between the PMed system and an external database, the PMed report was delayed until the Sunday.

The findings presented in both Table 5 and Table 6 provide critical information regarding the considerations that be need to be addressed in guiding the design of future canine PMed studies. Refining the logistics through the identification of the possible failure points in the process are important metrics that were addressed in the primary objective of this study. These findings will be used to design future PMed trials, with the expectant outcome being a reduced rate of attrition rate for the enrolled subjects. Moving forward, the study designs will also include a treatment phase that will rely upon the effective use of the PMed report by the Veterinarians. Therefore clinician feedback was captured regarding their impressions following the receipt of the PMed report for their patient(s). A deeper understanding of the clinicians thoughts and concerns related to the report presentation will assist in our understanding of how best to present the data to the clinician and support their decision making; with the ultimate aim of providing an informed drug prioritization schema to aid in their prospective treatment decisions. In general the PMed reports were well received and found to be easy to read and presented in an acceptable format. An example report for subject TL-141 is provided in the Additional file 2. Support for additional treatment based PMed trials based on the predictions provided in the PMed report was supported by an overwhelming 85% of clinicians, who stated they would consider using the report under the appropriate circumstances. This encouraging feedback, together with their constructive comments suggest that additional support and education regarding the information in the report and approaches to address drug availability, cost and canine dosing, would be critical factors in the implementation of a suitable therapeutic strategy based on the PMed reports.

## Discussion

Establishing a robust protocol, which is adaptable to the inherent challenges that can arise whilst working with clinical samples in real time, is critical to the success of any trial. In this report we have highlighted a protocol, and the challenges we faced, that will prove invaluable in the design of a prospective personalized medicine treatment trial in canines with osteosarcoma. Osteosarcoma was an excellent candidate tumor to study for number of reasons. Firstly, it is an extremely common disease in large breeds, with an incidence estimated to be around 13.9/100,000 [18]; these numbers will have a positive impact on the rapid recruitment of study participants. Secondly, although amputation and adjuvant chemotherapy have been shown to be extremely effective in the short term, the long term survival is poor and the current armamentarium for canine osteosarcoma are restricted to combinations of classical cytotoxic agents e.g. doxorubicin and platinum compounds (reviewed in [24]). Current therapeutic development for canine osteosarcoma involves the modification of current protocols and Standard of Care (SOC) agents with limited success [43], rather than the integration of new therapeutic agents as single or combinational therapy. Personalized medicine strategies provide an opportunity to expand a patient’s “therapeutic opportunities” by examining the molecular/biological factors that are fundamental to that individual’s disease etiology and progression. Using various bioinformatics methods described here that integrate both classical chemotherapeutics with a large library of molecularly targeted agents designed to inhibit intracellular targets, agents that block the drivers of the disease phenotype can be identified. At present, PMed approaches in veterinary oncology are limited to the administration of toceranib or masitinib in dogs with mast cell tumors containing c-kit mutations [44]. The translational value of canine osteosarcoma provides a vital opportunity to further refine the PMed approach through the application of drug predictions to treatment of naïve tumors in a clinical trial, an opportunity that is not possible in human trials. Finally, the generation of data that can be directly related to the corresponding human disease, due to the close similarity of OSA in both species at multiple levels, makes it an excellent translational model for evaluating the principles of personalized medicine.

Sampling and handling of canine OSA tumors provides a unique set of challenges. Firstly, the precise location of the tumor for sampling could have a significant effect on the sample quality, i.e. the heterogeneity in the proportion of tumor tissue versus normal, and the differing extents of necrosis. Standardization with strict QC/QA was therefore critical and addressed by providing site specific training to the veterinary surgeons, to assist in the identification of the most suitable, viable tumor tissue for collection. Care was taken to harvest samples that were free of necrosis and not overly lytic located along the non-mineralized periphery of the tumor. The presence of cortical bone in the samples was also a challenge that was faced in this study as this could impede the processing of both the formalin fixed and snap frozen tissue. Prior to paraffin embedding, the formalin fixed tissue was decalcified for 3 hours in a solution containing formic acid. The snap frozen tissue was initially treated as bone-free and embedded in OCT for sectioning; any tissue that did not section in the cryotome, was removed from OCT, ground in liquid nitrogen, followed by RNA extraction in Trizol. As such, it was impossible to make the pathology reads from the OCT sections above and below those utilized for RNA, and in these cases the formalin fixed tissue was used as an appropriate surrogate.

Using the work flows and processes described in this study, we have demonstrated that it is feasible to process canine osteosarcoma samples received from multiple clinical sites and distribute a molecularly-guided, personalized medicine (PMed) report within 5 business days (Table 6, Final Turnaround Mean = 5.08 ± 0.8 days) from the time of sample receipt. Seven of the 20 samples enrolled in the study were not submitted for Affymetrix GeneChip profiling due to failure of genomic or pathological QC (6/7) or transportation problems (1/7). Thirteen samples were successfully genomically profiled, of which 11 were distributed to the veterinary clinicians within the 5 business day target. 2 samples failed the 5 day turn around, one due to staff shortages, and the second due to a database access failure which stalled the generation of the PMed report. This feasibility study has highlighted a number of critical failure points in the logistics of producing a timely PMed report. Within a restricted time frame, pathological QC failure is the most challenging criteria to address, as this, in most cases, would require additional sampling of the tumor. Repeat sampling of the original tumor fragment could address the issues of heterogeneous normal tissue contamination and necrosis. RNA quality, primarily assessed through the determination of RIN number, was found to be the single largest cause of sample attrition. Our experience has identified that re-sectioning (deeper into the tissue fragment) and repeat RNA isolation can, in the majority of cases, produce higher quality RNA. The 5-business day time constraints did not provide sufficient time to re-address those samples with a low RIN or failed Pathology. A recommendation for a future PMed clinical trial would be to increase the turnaround time from 5 to 7 business days, thereby providing additional time to re-process samples as necessary. Additionally, the RNA RIN QC will be raised from ≥6 to ≥7, therefore only samples with a RIN of ≥7 will be shipped, this would address samples RIN QC failure at the external contract laboratory, resulting from degradation of the RNA most likely due to repeated freeze-thaw cycles after shipment. While these modifications will slightly increase the maximum time required for sample processing within the confines of a real-time clinical protocol, it should nonetheless significantly reduce the rate of attrition due to low quality RNA. With specific reference to this study, an improved quality of RNA would have the potential to increase the overall pass rate from 65% to 85%. Additionally, based on current canine osteosarcoma SOC clinical protocols for patients following amputation, there is considerable flexibility to permit an increase the turnaround from 5 to 7 business days. Adjuvant therapy usually commences following a 14 day surgical recovery period as it has been shown that there is no additional patient benefit to starting chemotherapy soon after surgery [45].

While the primary goals of the study were achieved and the pitfalls identified, the success of a prospective PMed clinical trial is dependent upon the commitment and active participation of the clinical veterinarians. To further improve the infrastructure necessary to support the clinicians and identify the specific challenges that will be faced while implementing adjuvant therapies, opinions were captured from the participating clinicians. Based upon the responses, the reaction to the PMed reports was overall positive, and useful information was provided that will be used to steer the development of a prospective clinical trial protocol in the future. As with human trials, the role of a multidisciplinary tumor board will be critical in advising the clinician as to the appropriate therapy(s). One particular challenge that will need to be addressed in future studies will be the lack of established canine dosing for the FDA approved medications identified through our PMed approach. This has been addressed in a cursory review (see Additional file 1: Table S1) although a more comprehensive evaluation is certainly warranted and will most likely involve a restricted drug list in which there is known canine use. Furthermore, a prospective PMed trial in which most suitable therapies are applied to the patients will need to offer drug reimbursement as an incentive to owners to enroll their companion pets.

## Conclusions

The data presented in this report demonstrate that it is possible to provide a PMed report to the veterinarian in 5 days from receipt of sample. This feasibility study has identified a number of areas of the protocol that can be enhanced to reduce the number of samples that fail the QC criteria established to maintain the integrity of the PMed predictions. Additionally, a number of weaknesses have been identified post-report distribution, which can be addressed to assist in the clinical interpretation and application of the PMed report towards selection of the most appropriate therapy. Moreover, while our current approach leverages molecular technologies and associated bioinformatics approaches for analysis of gene expression, the recent emergence of next-generation sequencing technologies holds additional promise of identifying additional genomic aberrations (mutations) within individual patient tumors that may provide a more complete depiction of the multiple facets which collectively comprise the cancer phenotype (Discussed in [46-48]). Whether these more advanced technologies, including the computational tools required to analyze and interpret the vast quantities of data, can be performed in a time and cost effective manner remains to be determined.

## Abbreviations

OSA: Osteosarcoma; PMed: Personalized medicine; H&E: Hematoxylin and eosin; QC: Quality Control; QA: Quality Assurance; CLIA: Clinical Laboratory Improvement Amendments; ACI: Animal Clinical Investigations; CRL: Clinical Reference Laboratory; RT: Room temperature; OCT: Optimal Cutting Temperature; FFPE: Formalin Fixed Paraffin Embedded; RIN: RNA integrity number; SOP: Standard Operating Procedure; VARI: Van Andel Research Institute; FDA: Food and Drug Administration; DEGs: Differentially expressed genes; FTP: File transfer protocol; PGSEA: Parametric Gene Set Enrichment Analysis; PCA: Principal Component Analysis; SOC: Standard of care; NSAIDS: Non-steroidal Anti-inflammatory Drugs; M: Male; MC: Male castrated; FS: Female spayed.

## Competing interests

The study was funded by Zoetis Inc. SGK, HS and KRM are Zoetis employees. CPW is a consultant for the company TransMed Systems and an inventor of the XenoBase technology that has been licensed to both TransMed Systems and Intervention Insights. All other authors have no competing interests.

## Authors’ contributions

NRM, DMC, SGK, HS, AWR, DC, KRM and CPW Conceived, designed and planned the study. NRM, HS, EE, MS and TG developed the methodology and collected the the normal canine samples. HS, AWR and DC directed the collection of study samples. NRM, DMC, SGK, HS, AWR, KRM and CPW analyzed and interpreted the data. RS performed the pathological analysis. DMC and MM performed the bioinformatics analysis (e.g., PMed report generation, statistical analysis, biostatistics, computational analysis): NRM drafted the manuscript. All authors read and approved the final draft.

## Supplementary Material

Additional file 1: Table S1 PMed Drug list reviewed for Canine use and dosing. Click here for file

Additional file 2 Example PMed Report for subject TL-141. Click here for file
