# Supplementary material for: A multi-site feasibility study for personalized medicine in canines with Osteosarcoma
Source: J Transl Med. 2013 Jul 1;11:158. doi: 10.1186/1479-5876-11-158 (PMC3702405; doi:10.1186/1479-5876-11-158)
Supplement: Additional file 2 — Example PMed Report for subject TL-141. [file 1479-5876-11-158-S2.pdf]

REPORT

Patient

Patient Name: PAHA61 AMP-2 TL-141 **Oncologist:** Dr. Tony Rusk VDM
Date of Birth: August 01, 2011 **Practice:** Animal Clinical Investigation LLC
MRN: 4926 Wisconsin Avenue, NW
Generated Date: 8/26/2011 Washington DC, 20016
Order ID: 7816-5523-0717-8311 **Report Version:** 1.3.0.0

Diagnosis: Long bones of lower limb, malignant neoplasm
Pathology Diagnosis: Osteosarcoma, NOS (C40._, C41._) (9180/3)
References Run: Canine_Reference_std

Comments: Term Osteosarcoma reference PAH normal

Results Summary

RANK	DRUG	METHOD	PEDIATRIC	REIMBURSEMENT	OTHER
				SUPPORTING INDUSTRY EVIDENCE	
1	amiloride	Drug Response Signatures (1 target)<br>Drug Target Expression (1 target)		0/25	24/1084
2	trazodone	Drug Target Expression (1 target)		0/25	3/1084
3	temsirolimus	Drug Target Expression (1 target)		0/25	5/1084
4	thioguanine	Drug Target Expression (1 target)		0/25	10/1084
5	paclitaxel albumin-bound	Biomarker-Based Rules – Sensitive (1 target)		0/25	0/1084
6	sirolimus	Drug Response Signatures (1 target)		1/25	44/1084
7	bortezomib	Biomarker-Based Rules – Sensitive (1 target)		1/25	11/1084
7	vorinostat	Biomarker-Based Rules – Sensitive (1 target)		0/25	15/1084
9	cisplatin	Drug Sensitivity Signatures (1 target)		21/25	851/1084
10	raloxifene	Network Target Activity (2 targets)		0/25	18/1084
11	colchicine	Drug Target Expression (1 target)		0/25	23/1084
11	vinblastine	Drug Target Expression (1 target)		2/25	71/1084
13	dutasteride	Drug Target Expression (1 target)		0/25	5/1084
13	finasteride	Drug Target Expression (1 target)		0/25	3/1084
15	axitinib	Drug Target Expression (1 target)		0/25	1/1084

Drug Method Comparison

RANK	SUMMARY METHOD 15 DRUGS	DRUG TARGET EXPRESSION 13 DRUGS	DRUG RESPONSE SIGNATURES 3 DRUGS	DRUG SENSITIVITY SIGNATURES 4 DRUGS	NETWORK TARGET ACTIVITY 3 DRUGS	NETWORK CONVERGENCE	NETWORK DIVERGENCE	BIOMARKER-BASED RULES – SENSITIVE 3 DRUGS
1	amiloride	trazodone	sirolimus	cisplatin	raloxifene			paclitaxel albumin-bound
2	trazodone	amiloride	amiloride	lomustine	raloxifene			bortezomib
3	temsirolimus	temsirolimus	digoxin	carmustine	tamoxifen			vorinostat
4	thioguanine	thioguanine		etoposide	yohimbine			
5	paclitaxel albumin-bound	colchicine						
6	sirolimus	vinblastine						
7	bortezomib	dutasteride						
8	vorinostat	finasteride						
9	cisplatin	axitinib						
10	raloxifene	sorafenib						

Detailed Results

RANK	DRUG	-LOG(P)	SELECTED BY MULTIPLE METHODS	BIOMARKER	DRUG MOLECULAR EVIDENCE	REIMBURSEMENT	OTHER
**Results Summary**							
1	amiloride	12.40					24
2	trazodone	10.00					3
3	temsirolimus	6.27					5
4	thioguanine	6.26					10
5	paclitaxel albumin-bound	5.85					
6	sirolimus	4.70				1	44
7	bortezomib	3.83				1	11
7	vorinostat	3.83					15
9	cisplatin	3.82				21	851
10	raloxifene	3.23					18
11	colchicine	3.04					23
11	vinblastine	3.04				2	71
13	dutasteride	2.99					5
13	finasteride	2.99					3
15	axitinib	2.72					1
15	sorafenib	2.72				1	13
15	sunitinib	2.72				1	8
18	lomustine	2.70					23
19	felodipine	2.60					6
19	nifedipine	2.60					33
21	carmustine	2.17				14	76
22	etoposide	1.70				17	296
23	tamoxifen	1.61					53
24	digoxin	1.40					18
24	yohimbine	1.40					2
**Drug Target Expression - Total Drugs: 13**							
1	trazodone	83.90		HRH1	Link		3
2	amiloride	9.61		SCNN1A	Link		24
3	temsirolimus	6.27		MTOR	Link		5
4	thioguanine	6.26		IMPDH1	Link		10
5	colchicine	3.04		TUBB2A	Link		23
5	vinblastine	3.04		TUBB2A	Link	2	71
7	dutasteride	2.99		SRD5A1	Link		5
7	finasteride	2.99		SRD5A1	Link		3
9	axitinib	2.72		KDR	Link		1
9	sorafenib	2.72		KDR	Link	1	13
9	sunitinib	2.72		KDR	Link	1	8
12	felodipine	2.60		CACNA2D1	Link		6
12	nifedipine	2.60		CACNA2D1	Link		33
**Drug Response Signatures - Total Drugs: 3**							
1	sirolimus	4.70				1	44
2	amiloride	2.78					24
3	digoxin	1.40					18

**Drug Sensitivity Signatures - Total Drugs: 4**							
1	cisplatin	3.82				21	851
2	lomustine	2.70					23
3	carmustine	2.17				14	76
4	etoposide	1.70				17	296
**Network Target Activity - Total Drugs: 4**							
1	raloxifene	1.61		ESR2	Link		18
1	raloxifene	1.61		ESR2	Link		18
1	tamoxifen	1.61		ESR2	Link		53
4	yohimbine	1.40		ADRA2B	Link		2
**Biomarker-Based Rules – Sensitive - Total Drugs: 3**							
1	paclitaxel albumin-bound	5.85		SPARC			
2	bortezomib	3.83		RAD23B		1	11
2	vorinostat	3.83		RAD23B			15
**Biomarker-Based Rules – Insensitive - Total Drugs: 1**							
1	doxorubicin	2.94		ABCC1		32	1269
